# A simple solution to the Rietveld refinement recipe problem

**DOI:** 10.1107/S1600576723011032

**Published:** 2024-02-01

**Authors:** B. H. Toby

**Affiliations:** a Argonne National Laboratory, 9700 S. Cass Avenue, 401/B4192, Lemont, IL 60439, USA; ESRF, France

**Keywords:** Rietveld analysis, powder diffraction, parameter selection, *GSAS-II*

## Abstract

Rietveld refinements are widely used for many purposes in the physical sciences. Conducting a Rietveld refinement typically requires expert input because correct results may require that parameters be added to the fit in the proper order. This order will depend on the nature of the data and the initial parameter values. A mechanism for computing the next parameter to add to the refinement is shown. The fitting function is evaluated with the current parameter value set and each parameter incremented and decremented by a small offset. This provides the partial derivatives with respect to each parameter, along with information to discriminate meaningful values from numerical computational errors. The implementation of this mechanism in the open-source *GSAS-II* program is discussed. This new method is discussed as an important step towards the development of automated Rietveld refinement technology.

## Abstract 2

A method to determine the order in which parameters should be added to a Rietveld refinement is presented.

## 1. Introduction

Rietveld analysis is the process where crystallographic models are directly fitted to powder diffraction data (Rietveld, 1969 ▸). Rietveld analysis has been a cornerstone of materials characterization for decades and is seeing increasing use for many research and process applications, including structure determination; characterization of materials properties such as texture and crystallite sizes; and quantification of the component amounts in multiphase samples in fields including chemistry, physics, geosciences, pharmaceuticals and engineering. On the basis of citations of the software, a minimum of several thousand Rietveld refinements are reported in the literature every year.

One of the more challenging aspects of Rietveld analysis is determining the order in which add parameters to the refinement. In addition to fitting crystallographic parameters, the refinement must also fit the background, lattice and peak shape parameters, and sometimes intensity correction terms, such as for texture, absorption or extinction (Toby, 2019 ▸). A crystallographer experienced with Rietveld refinement can look at the plot of the observed powder pattern, the computed pattern from the current model and their differences and from that graphic can tell at a glance which parameter(s) should be included next. However, transferring this knowledge to a novice is quite a challenge (Young, 1993 ▸). If an optimal ‘recipe’ is not followed, parameters may refine to unrealistic values. At this point it becomes unlikely that including additional parameters into the fit will allow the model to recover and find the true minimum. With older programs, refinements might even ‘blow up,’ where fitted values might exceed the computer implementation ranges for numbers and the software would fail. More modern minimization strategies, such as use of conjugate-gradient optimizers (Coelho, 2005 ▸), Levenberg–Marquardt and singular value decomposition Hessian modification decrease optimizer sensitivity to correlated parameters, but in the end, an accurate Hessian is still needed to determine the standard uncertainties for the fitted parameters. Other approaches, such as genetic algorithms, global optimization and Monte Carlo minimization have also been applied to powder diffraction, but more commonly for structure solution (David *et al.*, 2006 ▸; Padgett *et al.*, 2007 ▸; Pagola & Stephens, 2010 ▸; Mattei *et al.*, 2020 ▸; Habermehl *et al.*, 2022 ▸).

The difficulty inherent in parameter order selection was summarized nicely by Ozaki *et al.* (2020 ▸) who stated

‘It is commonly known that refining all parameters at once often leads to physically unreasonable results… it is not guaranteed… [to] lead researchers to the optimal crystal structure… Considering the wide use of Rietveld refinement… that only proficient experts can exploit Rietveld refinement properly, should be improved.’

In that work, these authors developed a ‘blackbox optimizer’ to drive the *GSAS-II* Rietveld code (Ozaki *et al.*, 2020 ▸; Toby & Von Dreele, 2013 ▸, 2023 ▸; O’Donnell *et al.*, 2018 ▸). They surveyed a number of machine learning optimization approaches, but selected a Bayesian methodology, in part due to its efficiency. However, it still requires that the refinement be performed at least several hundred times, rather than once, so it remains computationally expensive. Likewise, the *AutoFP* expert system allows for automation of *FullProf* (Cui *et al.*, 2015 ▸; Rodríguez-Carvajal, 1993 ▸). More recently, Szymanski *et al.* (2023 ▸) described a robotically enabled self-driving inorganic synthesis laboratory that includes an ‘automated approach to multiphase Rietveld refinement’ based on *GSAS-II*. Many of the refinement plots provided in that work appear as if they would benefit from further refinement progress, indicating that further work on automating refinements is still needed (Peplow, 2023 ▸).

Presented here is a direct and compact computational approach that can identify the next parameter(s) to be added to the refinement. This method has been implemented as an option within *GSAS-II*. This method is conceptually simple, should be easy to implement in other codes and uses relatively minimal computer time. It is envisioned as a step towards the development of fully automated Rietveld refinement tools.

## 2. The worst-fit parameter concept

**Figure 1:**
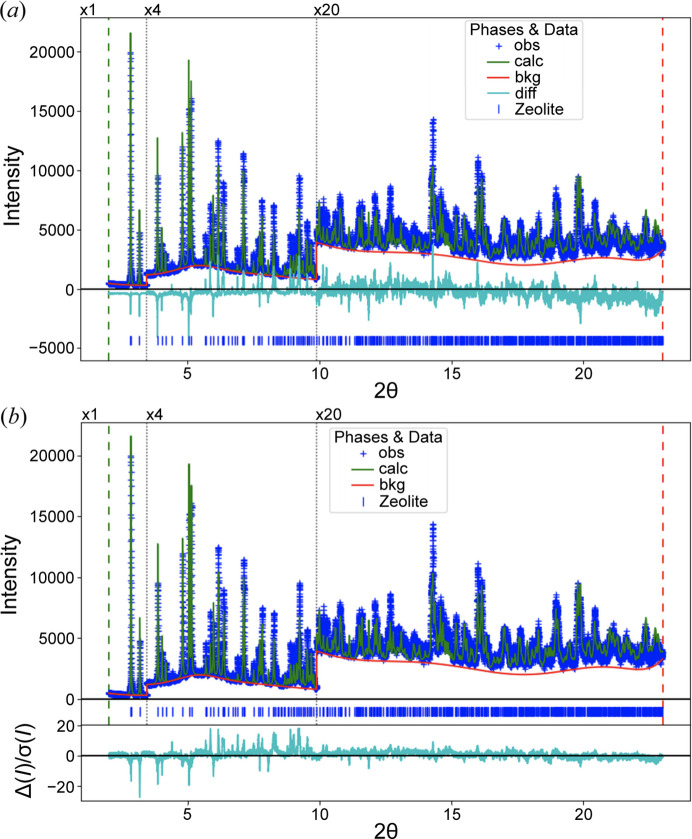
‘Rietveld plot’ showing the lack of agreement between observed powder diffraction data and those computed from a less than ideal crystal structure model. (*a*) Observed pattern shown with blue plus signs with the computed pattern superimposed as a green line. The lower cyan curve shows the difference between the observed and computed patterns. The red line shows the fitted background and the blue vertical lines show the reflection positions. (*b*) Similar plot, but the cyan curve shown with a separate vertical axis provides the difference between the observed and computed values divided by the standard uncertainty. A clearer view of the impact of these differences on the overall fit is seen in (*b*).

The key for determining the order in which add parameters to a refinement is a plot with the observed powder pattern, the computed pattern from the current model and their differences; this is sometimes called a Rietveld plot. The Rietveld plot shown in Fig. 1 ▸(*a*) demonstrates that, while the structure does provide a general match to the observed intensities, the model is incorrect in that it does not closely match the observed intensities. This visualization provides a simple way to access the quality of a fit in a single graphic, albeit one that should be viewed at multiple magnification scales. One of the great strengths of powder diffraction crystallography is that this plot provides a clear view of the fit quality, particularly since refinement metrics alone cannot be used as a guide to quality (Toby, 2006 ▸).

However, note that all minimization processes utilize some weighting of data. It has been shown that the optimal fit is obtained when observations are weighted by their experimental uncertainties (Prince, 2004 ▸). When these uncertainties are unknown or other weighting is used, the precision of the result is degraded, but the accuracy is not, unless the weighting were to accentuate some form of systematic error. The traditional Rietveld plot can be made more valuable if the difference values are plotted as the weighted differences, *i.e.* displaying the differences between the observed and computed points divided by the standard uncertainty for each point, as taken from the weight [Fig. 1 ▸(*b*)], rather than plotting the differences directly. Showing the fit relative to weighting has three advantages. First, it provides information on how the data are being weighted. Further, as intensities in the pattern increase, typically so do their uncertainties. The unweighted differences tend to accentuate deviations that occur in intense parts of the pattern, even though these differences may be statistically insignificant. Also, while the direct differences are on the scale of the diffraction intensities, which is an inherently arbitrary axis, when optimally weighted, differences have a statistical expectation value of unity, and thus the weighted differences are on a statistically well-defined absolute scale.

**Figure 2:**
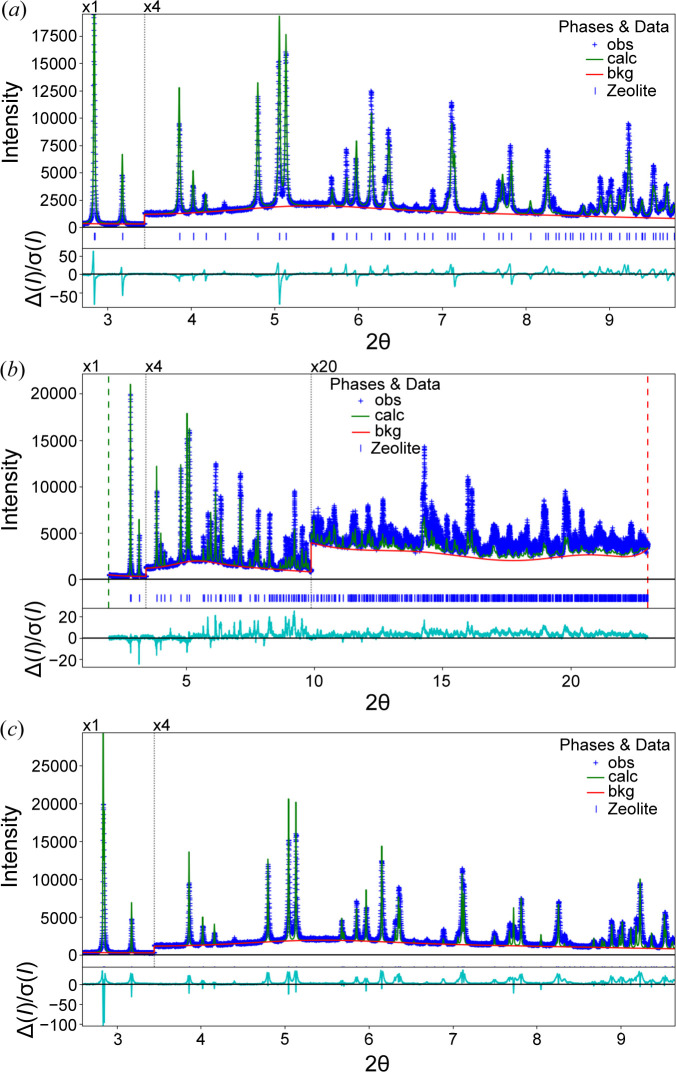
Visual comparison showing different misfits due to incorrect parameter values. Figure components follow the description for Fig. 1 ▸(*b*). (*a*) A lattice parameter is shifted, causing differences that are highlighed in the difference plot for some peaks. (*b*) The *U*_iso_ values are too large, causing the reflection intensities to fall off more quickly than expected. (*c*) The peak widths are misfitted; this is also visually apparent from the difference curve with features that differ from (*a*).

When an experienced crystallographer views a Rietveld plot, they look to see what is causing the greatest disagreement (McCusker *et al.*, 1999 ▸). If the observed peaks are shifted relative to the calculated peaks, the lattice parameters (or related instrumental corrections) are at fault. However, if all the intensities in either pattern are significantly larger than those in the other, for example, then the scale factor is not likely to be optimal. Alternatively, if the intensity agreement shows systematic deviations that vary as a function of *Q*, then the atomic displacement parameters (ADPs; typically *U*_iso_ values) are problematic. A few examples of this are shown in Fig. 2 ▸. If the deviations are for some reflections but not all, as is seen in Fig. 1 ▸, this is a likely indication of a problem that the atom positions of the model do not match the experiment; refinement of atomic displacement parameters may address this. In the case of the example in Fig. 1 ▸, there are as-yet unidentified inadequacies in the crystallographic model, so parameter optimization will not address this.

What the crystallographer attempts to determine visually from a Rietveld plot is the nature of the discrepancies between the observed data and the intensity values computed from the model. From that, one estimates which parameters are causing the greatest deviations between the data and the model. These parameters will be deemed the ‘worst fit.’ There may be a large number of parameters that are far from their optimum values, but the worst-fit parameters will have the largest impact on the overall agreement. Owing to parameter correlation, it may be impossible to optimize any other parameters before the differences due to these worst-fit parameters are addressed. Certainly, when the lattice parameters are not optimal, it makes no sense to attempt to optimize peak shape or structural parameters, and even background parameters may not refine well. Once the worst-fit parameter(s) have been fitted, some of the remaining unfit parameters will then become the worst fit and should be addressed next. While discerning the worst-fit parameters from visual features in a Rietveld plot is very likely a skill that a neural network could be taught, a relatively straightforward computational method will now be presented to determine the worst-fit parameters directly.

## 3. Computing the worst-fit parameter

In minimization problems, a factor described as χ^2^ is minimized. For Rietveld fitting, 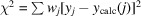 , where *y_j_* is the diffraction intensity for point *j*, *y*_calc_(*j*) is the calculated intensity for point *j* and *w_j_* is the weight for point *j*, where optimally 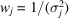 and σ_*j*_ is the standard uncertainty for *y_j_*. Note that if *y_j_* is an intensity in scaled counts, *y_j _* = *I_j_*/*n*, then 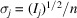 , where *n* is the scaling factor (unity for unscaled counts). For detection methods that do not count quanta, then optimal weighting requires that σ_*j*_ be estimated for the detector. With 2D detection, σ_*j*_ can be estimated from the intensity spread in nominally equivalent pixels. Note that χ^2^ here should not be confused with the quality metric, the reduced χ^2^ = 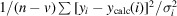 , where *n* and *v* are the number of observed data points and the number of refined parameters, respectively. In single-crystal refinements, the term GOF or goodness of fit is used, where the square of the GOF is equivalent to the reduced χ^2^. The reduced χ^2^ metric will be unity with an ideal model because the statistical expectation value for 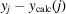 is the definition of σ_*j*_.

How a function responds as a parameter is changed is, by definition, the partial derivative of that function with respect to the parameter. The sign of that partial derivative indicates if increasing or decreasing the parameter improves the fit. If we evaluate 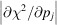 at the values for all parameters in our current model, *p_j_*, the one where the magnitude of the derivative is largest should be the one that will have the largest impact on minimizing χ^2^, but, as will be discussed, other considerations will be needed due to the discrete numerical computation to be done here. Note that the sign of the offset to be applied to a parameter is not relevant for our purpose, which is only to determine which parameters will have the largest effect on χ^2^ if the parameter is varied. The Rietveld minimizer (traditionally a variant on least squares) will determine both the magnitude and the sign of the shift to be applied to each parameter as additional parameters are included in the refinement.

To consider how this works in practice, note that the scale factor for a dataset will multiply every point in the computed diffraction pattern. One can expect the partial derivative of χ^2^ with respect to the scale factor to be quite large, except when the scale factor has been exactly minimized. Likewise, background values are subtracted from every point in the pattern and will also very significantly affect χ^2^. When away from the best-fit value, either the scale factor or the background values will almost certainly be the ‘worst-fit parameters’ since the former has a large impact on the agreement for every peak in the pattern and the latter will affect every point. This is why, if one writes a naive prescribed parameter order ‘recipe’ for Rietveld, the scale factor or background are almost always the first parameters to be minimized. Once those have been fitted, one can advance to other parameters. Note that this assumes that the unit-cell parameters are fairly close to correct values, so that there is appreciable overlap between the observed and computed peaks. If there is no significant overlap between the observed and computed peaks, neither the initial scale factor nor the cell constants are likely to refine to better values. On the other hand, if the cell parameters provide some peak overlap but are far from optimal, optimization of the unit cell is needed before the scale factor can be properly minimized.

With the scale factor and background fitted, the next parameter to be fitted will depend on how the observed and calculated patterns differ. This may be where the unit-cell parameters need to be added. If the cell parameters do agree well with the observed data, but the peak widths do not, it may be necessary to treat the microstrain or crystallite size before additional refinement progress can be made.

Returning to the calculus, if a function is fully minimized, the first derivative for all parameters is zero and the second derivative is positive, 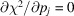 ; 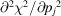 > 0. Note that these statements are only true if the model is continuous and the derivatives are evaluated analytically, meaning that computation accuracy is essentially infinite. However, for crystallographic fitting, we evaluate χ^2^ with numerical computations and with discretely observed data points. This means that we have finite precision in these computations. As an example for how this affects computations, consider the partial derivative for the scale factor. If a least-squares minimization cycle has been applied, then it will be at the ‘correct’ minimum, at least for all other parameter values held at their current values. This parameter is linear, so least squares is not an approximation; it usually converges quickly. However, these computations are still not exact. When we describe a parameter as having converged, we mean that the parameter is still showing shifts in optimization cycles, but the shifts are less than the standard uncertainty of that parameter. In fact, any two values for a parameter that are separated by less than two times the standard uncertainty of that parameter are considered indistinguishable. Taking the result from the minimization and computing 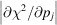 for the scale factor is likely to give a large value even when this parameter has been properly minimized because, as noted previously, the χ^2^ function is extremely sensitive to the scale factor. Even when the parameter has converged to the point where shifts are at insignificant levels, very small differences due to roundoff error in the numerical computation can still allow for a very large derivative. So, alas, evaluating these derivatives for the current parameter set will not allow identification of the worst-fit parameters.

**Figure 3:**
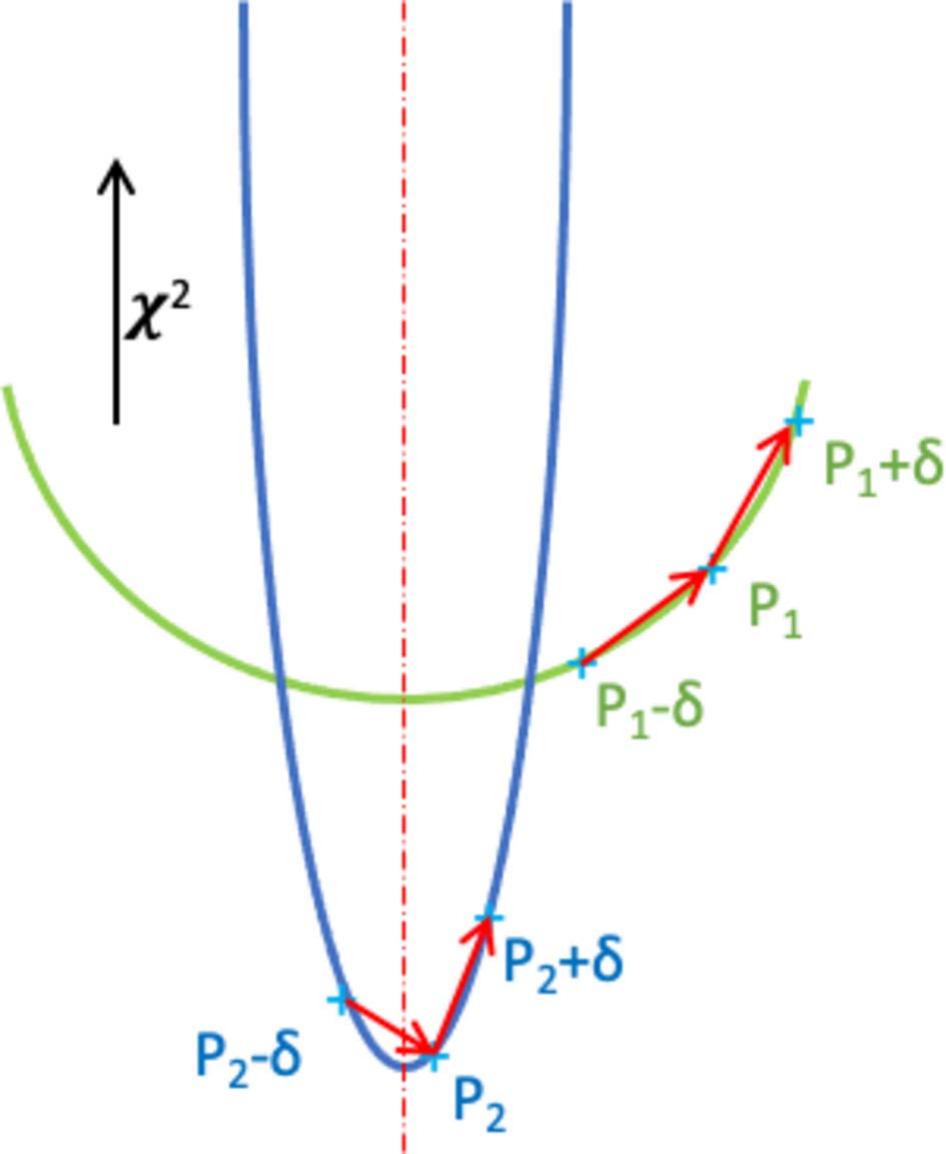
Graphical representation of how changes for two different parameters affect the fit. The vertical direction shows the relative change in χ^2^ as the parameter value is changed. The true minimum is indicated with a vertical dashed line in red; the first derivative computation is the slope of the lines shown as red arrows. These are obtained numerically from differences in the fitting function, χ^2^, evaluated at two locations. In the case of one parameter, shown in blue, point p_2_ is close to the minimum, but the fit is very sensitive to this parameter and thus the derivative can be quite large even though no significant improvement is expected from further minimization. The second parameter, in green, point p_1_, is far from its minimum. Note that the deriviates for the first parameter have the opposite sign while those for the second parameter have the same sign.

Informed by the second derivative, which examines how 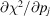 changes as *p_j_* changes, we can discern the worst-fit parameters from those that have a large first derivative due to computational inaccuracies, despite being well minimized. If for parameter *p_j_* we evaluate the partial derivative at locations *p_j_ − δ* and *p_j_ + δ*, where δ is a small perturbation to *p_j_*, on the order of or less than the standard uncertainty on that parameter, we can test if the minimum is between *p_j_ − δ* and *p_j_ + δ*. If the minimum value for the parameter is well removed from the current value, *p_j_*, then we would expect 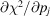 to be about the same when evaluated at the three values *p_j_ − δ*, *p_j_* and *p_j_ + δ*. On the other hand, if the current value *p_j_* is already close to the minimum, then we would expect 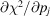 to have opposite sign when evaluated at *p_j_ − δ* and *p_j_ + δ*, indicating that the second derivative is zero somewhere in that range. If this is true, we would not expect to see a significant improvement in the fit by optimizing that parameter. We would also expect to see these opposite signs if near a *maximum* for χ^2^ with respect to *p_j_*, but since we must start a fit with parameter values that are close to the correct model, we would not expect to encounter a local maximum in χ^2^. Fig. 3 ▸ illustrates this derivative computation process graphically. Thus, by computing the partial derivative at two locations, and requiring that 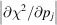 be large near the current value of *p_j_* and that 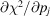 have same sign when evaluated at *p_j_* − δ and *p_j_* + δ, we can discern the parameter(s) that are the worst fit.

This computation has been implemented in the *GSAS-II* software suite, with details provided in Appendix *A* [App appa], as it is hoped that the discussion in that section and the accessibility of the source code will facilitate incorporation of this capability into other Rietveld codes. When invoked, the derivatives are computed for all appropriate parameters in the model, and those parameters where both derivatives have the same sign are reported to the user in a table, ordered so that the largest-magnitude derivative (the worst-fit parameter) is reported first. The time needed for this process will depend greatly on the number of computed reflections, the diffraction points in the pattern(s) and the number of parameters in the model, but a computation time on the order of seconds to minutes is likely.

## 4. Discussion and conclusions

This work has presented a tool that offers novice crystallographers a mechanism to determine the order in which parameters should be added into a Rietveld analysis. This capability is easily added to a Rietveld code and herein we outline how this can be done. Access to the Rietveld engine source code may not even be needed. A script could be written to modify the input parameters supplied to a compiled Rietveld code. The required partial derivatives could then be accumulated from the resulting χ^2^ values.

Nonetheless, despite this advance, powder diffraction crystallographers will still need to understand the meaning of the parameters and how they interact with respect to changing the agreement between the data and the computed pattern. The method presented here will identify the parameters in the model that will offer the largest change in χ^2^, but not all of these parameters are appropriate to vary. For example, with the model presented in Fig. 2 ▸(*c*), where the peak shapes are not well fitted, a much larger derivative is seen for an instrumental broadening term than for sample broadening, but the latter is a more appropriate term to include in the model.

What has been presented here, or, for that matter, the work of Ozaki *et al.* (2020 ▸), addresses the serious problem of the order to introduce parameters into a model seen in many contemporary crystallographic refinement codes. This problem may be less acute for Rietveld implementations with more robust minimization strategies (Coelho, 2018 ▸), but it has not been established whether the order that the parameters are introduced remains important. Addressing this problem still leaves several other significant tasks that at present still require the attention of an experienced crystallographer: this is determining how a model should be parameterized, such as what intensity correction terms are appropriate for the measurement. A lack of useful observables (Sivia, 2000 ▸) may prompt introduction of constraints, such as grouping the ADP values for similar atoms or use of geometrical constraints, such as a rigid body, or similarly use of compositional or geometric restraints. These constraints and restraints change how the overall fit responds to changes in the parameters. Likewise, the crystallographer must also decide when to expand the model description, for example, to treat anisotropic peak broadening, or when the data are insufficient to support full parameterization; for example, limited data range may prevent simultaneously fitting crystallite size broadening and microstrain. In those cases, on the basis of information on the sample origins, a choice must be made as to which parameter provides a more sensible model. The quality of a fit must also be determined from the validity of the results, so an expert system that truly automates refinement must not only determine parameterization but also judge the physical and chemical plausibility of the refined parameters. Thus, there is considerable work that remains before Rietveld analysis can be made automatic, but it is now possible to envision automating parameter introduction, even if the analyst must specify considerable information based on the measurement type(s) and the family of materials. Likewise, it becomes possible to imagine software that performs automatic testing of subgroup and supergroup structures or even helps wade through the wealth of models for background, peak shape and intensity correction factors that add so much complexity to Rietveld analysis. Nonetheless, even if Rietveld analysis were to be automated, an even more difficult problem is raised by the example in Fig. 1 ▸, where the model clearly shows significant agreement with the data but, even with all parameters optimized, still inadequately fits the data. Parameter optimization will not address this. Additional models must be developed and explored. This at present very much depends on the imagination and experience of the crystallographer.

## Appendix A Implementation in *GSAS-II*

In this section, specific *GSAS-II* routines are discussed. Further documentation on those routines, as well as a listing of their source code, can be found in the code developer documentation, available as web pages or for download as portable (PDF or Epub) documents (Toby & Von Dreele, 2023 ▸). Note that, while *GSAS-II* computes analytic derivatives for most varied parameters as part of the refinement process, the code for that is embedded into assembly of the Hessian matrix, which makes it difficult to access in other contexts. Since the derivative computation needed here is performed at two points for each parameter and since the analytic derivative computation takes a similar amount of time to compute as does evaluation of χ^2^, computation of numerical derivatives does not take appreciably more time than analytic derivatives would. Analytic derivatives would likely be somewhat more precise than numerical derivatives, but since these derivatives are only used to identify parameters that have significant leverage over χ^2^, this precision is not needed.

The computation of the ‘worst-fit’ parameters is performed in the *GSAS-II* graphical user interface from an entry in the ‘Calculate’ menu labelled ‘Parameter Impact’. This invokes the method OnDerivCalc() in class GSASIIdataGUI.GSASII, which in turn invokes the refinement routine, GSASIIstrMain.Ref ine(), but with a special argument that indicates that partial derivatives should be computed without completing a refinement. This Ref ine() routine initially constructs a Python dict data structure with all the parameter values, as well as assembling the powder data and other information needed for computation of χ^2^ in the routine GSASIIstrMath.errRef ine(). Note that the parameter dict is keyed by a variable name that uniquely identifies the role the parameter has in the model computation and the value corresponding to the dict entry is the numerical value of the parameter. There are some entries in this data structure that cannot be varied sensibly. For example, any parameter in that dictionary that does not have a floating point value is ignored. In the case of lattice parameters, the unit-cell symmetry is used to determine which of the reciprocal cell tensor entries should be included.

The process for computing partial derivatives for all appropriate parameters is done in routine GSASIIstrMain.AllPrmDerivs(), where the value of χ^2^ is evaluated with the current parameter set and saved, to be used later in the evaluation of the partial derivative for each parameter. We will label this here as χ^2^(0). Next, there is a loop over appropriate parameters (noting that some entries in the dict are not appropriate to be changed). The parameter being considered will be labelled *p_j_*. An offset to be applied to that parameter, *δ_j_*, is determined by the role that parameter has in the model. Note that, if the value for *δ_j_* is too small, the derivative computation will be dominated by roundoff errors and will be inaccurate. If *δ_j_* is too large, it will not sample the derivative in the vicinity of the current value for that parameter. For most parameters used in *GSAS-II*, *δ_j_* is set to 10^−6^ or the value of the parameter ×0.0001, whichever is larger. For fractional coordinates, δ*_j_* is set to 10^−6^, whereas for ADPs 10^−5^ is used. A few parameters related to sample positioning usually have values greater than one and for these *δ_j_* is set to 0.1. There may be a need to further improve these assumptions as more experience is gained.

For each appropriate parameter, χ^2^ is evaluated using GSASIIstrMath.errRef ine() with the selected parameter set to values of *p_j_* − δ and *p_j_* + δ. The χ^2^ values are labelled here as χ^2^(*p_j_* − δ*_j_*) and χ^2^(*p_j_* + δ_*j*_), respectively. Note that these computations are completely independent and could thus be distributed to different CPUs if speed via parallelization is desired. From the three χ^2^ values, three approximate partial derivative values are computed:   ; and the signs for the partial derivatives above and below the current parameter values are determined from  and  . These values are assembled into a dict that is returned by AllPrmDerivs() and after sorting are displayed in a table.

This capability has also been introduced into the *GSAS-II* scripting mechanism (O’Donnell *et al.*, 2018 ▸). In this case, the method ComputeWorstFit() is provided for the *GSAS-II* project class (G2Project). This method calls GSASIIstrMain.Ref ine() and returns a list of parameters, sorted by their derivative values, as well as a tuple with the values  ,  and  .
